# The attention network changes in breast cancer patients receiving neoadjuvant chemotherapy: Evidence from an arterial spin labeling perfusion study

**DOI:** 10.1038/srep42684

**Published:** 2017-02-17

**Authors:** Xingui Chen, Xiaoxuan He, Longxiang Tao, Huaidong Cheng, Jingjing Li, Jingjie Zhang, Bensheng Qiu, Yongqiang Yu, Kai Wang

**Affiliations:** 1 Department of Neurology, The First Affiliated Hospital of Anhui Medical University, Hefei, China; 2 Department of Medical Psychology, Anhui Medical University, Hefei, China; 3 Collaborative Innovation Centre of Neuropsychiatric Disorders and Mental Health, Anhui Province, China; 4 Center for Biomedical Engineering, University of Science and Technology of China, Hefei, China; 5 Department of Radiology, The First Affiliated Hospital of Anhui Medical University, Hefei, China; 6 Department of Oncology, The Second Affiliated Hospital of Anhui Medical University, Hefei, China; 7 Department of Breast Surgery, The First Affiliated Hospital of Anhui Medical University, Hefei, China

## Abstract

To investigate the neural mechanisms underlying attention deficits that are related to neoadjuvant chemotherapy in combination with cerebral perfusion. Thirty one patients with breast cancer who were scheduled to receive neoadjuvant chemotherapy and 34 healthy control subjects were included. The patients completed two assessments of the attention network tasks (ANT), neuropsychological background tests, and the arterial spin labeling scan, which were performed before neoadjuvant chemotherapy and after completing chemotherapy. After neoadjuvant chemotherapy, the patients exhibited reduced performance in the alerting and executive control attention networks but not the orienting network (*p* < 0.05) and showed significant increases in cerebral blood flow (CBF) in the left posterior cingulate gyrus, left middle occipital gyrus, bilateral precentral gyrus, inferior parietal gyrus, supramarginal gyrus, angular gyrus, precuneus, cuneus, superior occipital gyrus, calcarine cortex, and temporal gyrus (*p* < 0.01 corrected) when compared with patients before chemotherapy and healthy controls. A significant correlation was found between the decrease performance of ANT and the increase in CBF changes in some brain regions of the patients with breast cancer. The results demonstrated that neoadjuvant chemotherapy influences hemodynamic activity in different brain areas through increasing cerebral perfusion, which reduces the attention abilities in breast cancer patients.

An increasing body of evidence demonstrates that breast cancer patients suffer from many cognitive impairments related to adjuvant chemotherapy, including problems with verbal and/or visuospatial memory, attention deficits, difficulty in learning, confused thought processes^1^,^2^, and impairments in executive functions; these difficulties are commonly referred to as “chemobrain”^3^. Such cognitive impairment could have a significant effect on the patient’s quality of life, and thus, patients, physicians, and researchers should be more considerate of this possibility.

As a major component of the cognitive system, the attention work is involved in the centralization of brain or mental activities and the allocation of psychological resources^4^. Posner and Petersen proposed the attention network theory, which divides the attention systems into three distinct brain networks, that is, the alerting, orienting, and executive networks^5^. We recently reported findings from a cross-sectional neuropsychological study that the chemotherapy-treated breast cancer patients had significant impairment of attention networks^6^. Moreover, neuroimaging studies have identified the structural and functional changes that are associated with chemotherapy, including changes in gray and white matter^7^,^8^,^9^ and cerebral activation during performance of cognitive tasks^10^,^11^. Although previous reviews and our findings have confirmed this chemotherapy-induced cognitive impairment, the neural mechanisms underlying these deficits are still unclear.

In recent years, arterial spin labeling (ASL) has become an important noninvasive functional magnetic resonance imaging technique that uses magnetically labeled arterial blood water as an endogenous contrast agent to measure resting CBF images. This method has been widely applied to assess the cerebral perfusion in various clinical conditions, including neurodegenerative diseases^12^, age-related cognitive impairments^13^ and psychiatric disorders^14^. As such, ASL is a promising technique to assess whether therapy-induced cerebral perfusion changes could explain the cognitive impairments in patients with breast cancer after chemotherapy. To our knowledge, only one ASL study investigated chemotherapy-induced cerebral perfusion changes^15^. This study found that chemotherapy-treated patients had significantly increased post-treatment perfusion in the right precentral gyrus, which was correlated with the baseline of overall neuropsychological performance. However, in this study, some patients with breast cancer had received surgery and anti-estrogen therapy and were treated with different chemotherapeutic agents for systemic chemotherapy. Previous studies have shown that anti-estrogen therapy may impair cognitive function and may induce cerebral blood flow and metabolism alteration^16^,^17^. Different cognitive dysfunctions were also observed in patients who were treated with a variety of chemotherapy regimens^18^. Additionally, drug anesthesia of surgery can induce cognitive dysfunctions^19^,^20^ and cerebral perfusion change^21^. Therefore, it is necessary to exclude these confounding effects to determine whether the changes in cerebral perfusion and cognitive function are the result of the chemotherapy itself.

Due to the cross-sectional design used in our previous study, attention was not measured in the patients before their chemotherapy treatment. In the current study, patients with breast cancer were used to assess the potential changes in attention function before and after exposure to neoadjuvant chemotherapy in which the term “neoadjuvant” refers to the chemotherapy given before surgery, radiation, and anti-estrogen treatment, which offers advantages in terms of adding prognostic information and improving surgical options. Moreover, we used ASL to measure longitudinal differences in cerebral perfusion in these patients. Based on findings from the literature and our previous study, we hypothesize that after neoadjuvant chemotherapy, breast cancer patients have impaired performance in attention and changes in cerebral perfusion compared to themselves before neoadjuvant chemotherapy and healthy control participants. We also examined the relationships between the cerebral perfusion alterations and attention function changes that were observed in these patients.

## Results

A total of 68 participants meeting the criteria were initially enrolled in the study. One patient did not complete the follow-up assessment. Two additional participants (one patient and one healthy control) were excluded from the analysis because of excessive head motion artifact during data acquisition. Therefore, the final analytical sample size was 31 patients and 34 healthy controls.

### Participant demographics

Participant demographic and clinical information is shown in Table 1. All participants were premenopausal women between 28 and 52 years old. At the beginning of the chemotherapy treatment, the patients did not differ from the healthy controls with regard to age, education, depression, and fatigue score, with the exception of the anxiety score of the Hamilton Anxiety Rating Scale (HAMA). The pre-treatment patients had higher scores than the healthy controls, but these scores were below the cut-off value (HAMA scores <7). After receiving chemotherapy treatment, the patients had higher Cancer Related Fatigue (CRF) scores, but they did not have fatigue symptoms, and these scores were clearly below the cut-off value.

### Neuropsychological background tests

At baseline, there were no significant differences between the different cognitive domains of patients with breast cancer and healthy controls. Paired *t-*tests showed that post-treatment patients performed significantly worse in attention and concentration tests (WAIS Digit Span backward), memory (Delayed recall and Recognition test), and executive function (the Stroop Word, Stroop Interference test, Trail Making Test B, and Verbal fluency) when compared with pre-treatment patients (see Table 1). Compared with the healthy controls, the post-treatment patients also had significantly worse performance in the aforementioned cognitive domains.

### The three networks of ANT

Compared with the healthy controls, the patients showed no significant differences in the three networks before treatment but had a significantly shorter mean altering network reaction time (RT) and a higher mean executive control network RT after treatment. Paired *t-*tests showed that the post-treatment patients had a significantly shorter mean altering network RT and a higher mean executive control network RT than that of the pre-treatment patients. Additionally, we found that the overall mean RTs of the post-treatment patients were significantly longer than that of the pre-treatment patients and healthy controls. For this task, to eliminate this influence of the overall mean RTs, ratios can be used to examine the specific effects of the three networks. For each subject, the median RT in each condition was divided by the subject’s overall RT. The network ratio scores of the breast cancer patients and healthy controls are presented in Table 2. There were significant differences in alerting and executive control effects but not in the orienting effect for the pre- and post-treatment patients. We also found a significant difference in the alerting effect between the post-treatment patients and the healthy controls. No significant differences were found in the ratio scores for the three networks between the pre-treatment patients and healthy controls.

### Chemotherapy effects on perfusion change

**Figure 1:**
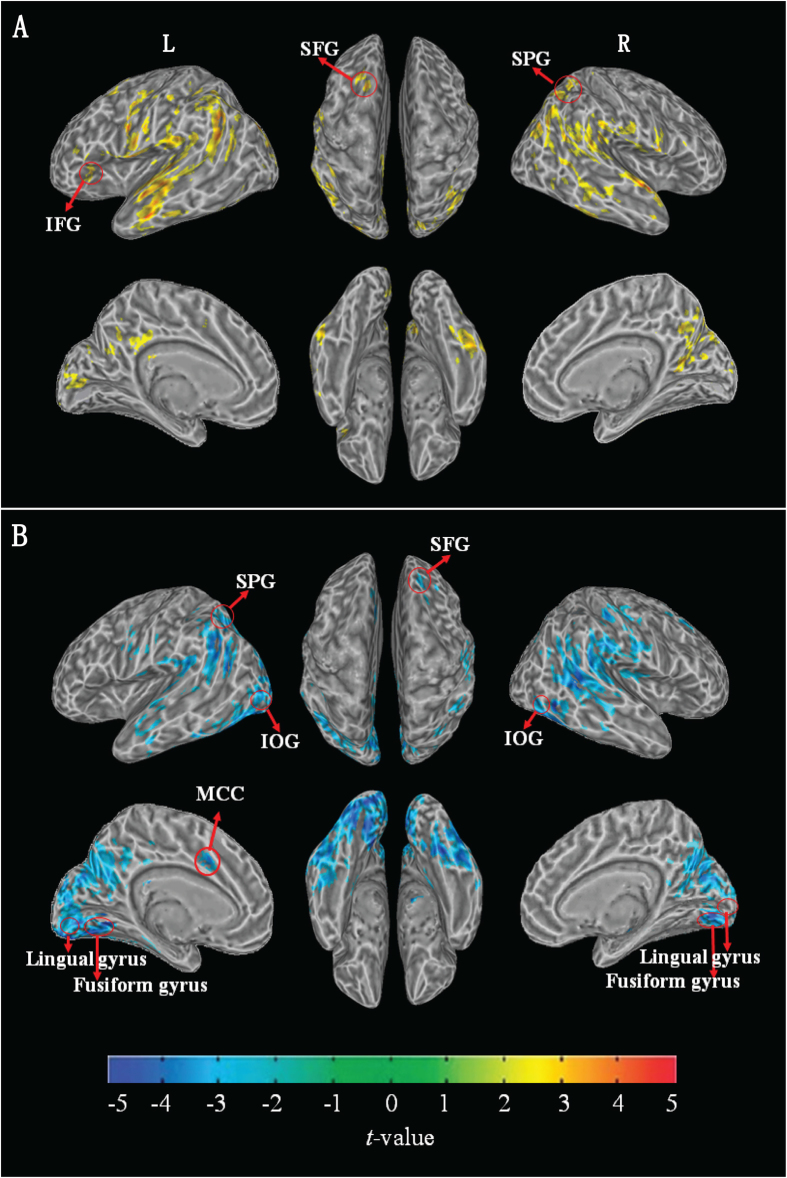
(**A**) Demonstration of results of intragroup comparison between pre- and post-treatment patients. The results were corrected using FDR correction, with q < 0.01, p < 0.0027 and a minimum cluster size of 100. Patients with post-treatment have greater perfusion than patients with pre-treatment in yellow brain areas. Significant perfusion changes of intergroup comparison between healthy controls and post-treatment patients were not observed in brain regions with labeled red circle. (**B**) Demonstration of results of intergroup comparison between healthy controls and post-treatment patients. The results were corrected using FDR correction, with q < 0.01, p < 0.0046 and a minimum cluster size of 100. Healthy controls have lesser perfusion than post-treatment patients in blue brain areas. Significant perfusion changes of intergroup comparison between pre- and post-treatment patients were not observed in brain regions with labeled red circle. (IFG: inferior frontal gyrus, SFG: superior frontal gyrus, SPG: superior parietal gyrus, IOG: inferior occipital gyrus, MCC: middle cingulate gyrus, L: left, R: right).

As shown in Table 3, the main effects of chemotherapy treatment were predominantly found in the left superior frontal gyrus, left posterior cingulate gyrus, left inferior frontal gyrus, right superior parietal gyrus, left middle occipital gyrus, bilateral inferior parietal gyrus, superior occipital gyrus, temporal gyrus, precentral gyrus, supramarginal gyrus, angular gyrus, precuneus, cuneus, and calcarine cortex (see Table S1 and Fig. 1A). The post-treatment patients exhibited an increase in CBF in all of the above brain regions. In the voxelwise independent two-sample t-test analysis, the post-treatment patients exhibited an increase in CBF in all of the above brain regions except for the left superior frontal gyrus, left inferior frontal gyrus, and right superior parietal gyrus. Additionally, increased CBF of the right superior frontal gyrus, left middle cingulate gyrus, left superior parietal gyrus, bilateral inferior occipital gyrus, lingual gyrus, and fusiform gyrus was observed in the post-treatment patients (see Table S2 and Fig. 1B) when compared with the healthy controls. No significant CBF differences were observed between the pre-treatment patients and healthy controls.

### The correlation between changes in perfusion and the attention networks

To identify the significance of the perfusion increase and the mechanism of attention deficits, we performed a correlation analysis between the perfusion change and attention performance. We calculated the change in CBF values (CBF at post-treatment minus CBF at pre-treatment) in the aforementioned significantly increased regions. Likewise, the post-treatment scores minus the pre-treatment scores of the attention networks were calculated to indicate changes in the attention networks. Significant correlations were observed between the changes in the alerting network score and the changed CBF values in the left posterior cingulate gyrus (r = −0.452; p = 0.014), bilateral inferior parietal gyrus (r = −0.550; p = 0.002), bilateral middle temporal gyrus (r = −0.381; p = 0.042), bilateral precuneus (r = −0.483; p = 0.008), and bilateral cuneus (r = −0.464; p = 0.011). Additionally, significant correlations were also found between the changes in the executive control network score and the changed CBF values in the left posterior cingulate gyrus (r = 0.680; p < 0.001), left superior frontal gyrus (r = 0.578; p = 0.001), left inferior frontal gyrus (r = 0.608; p < 0.001), bilateral inferior parietal gyrus (r = 0.507; p = 0.005), bilateral superior temporal gyrus (r = 0.548; p = 0.002), bilateral middle temporal gyrus (r = 0.601; p = 0.001), bilateral inferior temporal gyrus (r = 0.629; p < 0.001), bilateral precuneus (r = 0.654; p < 0.001), and bilateral cuneus (r = 0.567; p = 0.001).

## Discussion

To the best of our knowledge, our study is the first to investigate potential neoadjuvant chemotherapy-induced changes in the attention network in combination with cerebral perfusion changes, as indicated using ASL measures. Our results demonstrate impairment of the alerting and executive control attention networks and an increase in perfusion in brain regions after chemotherapy in patients with breast cancer. We found that after chemotherapy, the patients exhibited reduced performance in the alerting and executive control attention networks, and showed a significant increase in CBF of brain regions as shown above, when compared with patients before chemotherapy and healthy controls. In contrast, these differences were not present in either the patients before chemotherapy or the healthy controls. In addition, the patients performed worse on neuropsychological background tests from baseline to post-treatment. Furthermore, we found that such a decrease in the performance of the ANT task was significantly correlated with an increase in the CBF changes in different brain regions in the patients with breast cancer. Taken together, these results demonstrate that the neoadjuvant chemotherapy-induced cerebral perfusion changes may account for the attention deficits of the patients with breast cancer.

Pre-operative systemic therapy with AC (doxorubicin/cyclophosphamide) followed by pre-operative T (docetaxel) is a commonly used regimen for the neoadjuvant chemotherapy treatment of breast cancer. The individual agents of this regimen could induce cognitive changes via several processes that influence brain structure and function, such as oxidative stress, DNA damage, altered hormone levels, and immune response deregulation^22^. Several previous studies have shown that docetaxel and doxorubicin have peripheral anti-angiogenic effects that are associated with vascular toxicity^23^,^24^. This anti-angiogenic effect may be due to a disruption in the balance of peripheral-released cytokines and chemicals, which also cross the blood brain barrier to cause the veins to expand, ultimately resulting in indirect cerebral effects. Therefore, it is possible that the neoadjuvant chemotherapy-induced cerebrovascular changes could transform the hemodynamic response. Previous studies showed that lower CBF was related to poorer cognitive function^25^,^26^. Cognitive function would be improved after cerebral perfusion improvement^27^. The increase in the CBF in brain regions would compensate for cognitive function. However, based on our aforementioned findings, it is likely that the increase in cerebral perfusion is an unsuccessful compensatory mechanism that leads to cognitive impairments, in particular, the impairment of attention networks in patients with breast cancer.

According to Posner’s dominant theory of attention network, which emphasizes the involvement of neural attention networks in alerting, orienting, and executive control networks. Clinical studies and neuroimaging evidence indicate that the alerting system is associated with the frontal and parietal cortices and thalamus and that prefrontal cortex has a central role in supporting the executive control network^28^,^29^. The results of the present study showed that after chemotherapy, the patients exhibited reduced performance in these two networks and significant increases in CBF in the left superior frontal gyrus, left inferior frontal gyrus, bilateral inferior parietal gyrus, and temporal gyrus when compared with patients before chemotherapy and/or healthy controls. This behavioral performance change in attention networks is consistent with our previous cross-sectional study findings. Functional MRI studies report that breast cancer patients prior to neoadjuvant chemotherapy treatment had increased activations during working memory tasks compared to controls in areas such as the frontal gyrus^30^,^31^, insula, thalamus^31^. A possible explanation for these findings is that before neoadjuvant treatment, breast cancer patients are engaging in compensatory hyperactivation of brain neural circuitry to support working memory function in response to effects of the cancer disease^30^. Interestingly, the similar pattern has been found in this study. As we have discussed above, breast cancer patients exhibited increases in CBF in different brain areas after chemotherapy. The patients continue to maintain this hyperperfusion, likely as a result of cognitive changes, because we observed decreased cognitive performance was associated with the increases in CBF. On the basis of the discussion above, it is plausible to presume that neoadjuvant chemotherapy affects brain functions and cognition through its over-compensatory cerebral hyperperfusion in breast cancer patients.

The frontal and parietal lobes play a more direct role in regulating the focus of attention function, as previously shown. Furthermore, the posterior cingulate gyrus, precuneus and temporal cortex play an important role in attentional processing through organizing corresponding brain networks, such as the attention network, frontoparietal network, and default mode network (DMN), to help guide attention tasks^32^. Hosseini *et al*. revealed that patients with chemotherapy showed an altered functional connectivity in the right frontoparietal and left superior frontal gyri networks^33^. Decreased connectivity was also observed after chemotherapy in regions of the dorsal attention network that are involved in attention tasks^34^. The posterior cingulate cortex (PCC) and precuneus are the main hubs of the DMN, which consists of the PCC, precuneus, medial frontal, middle temporal, and lateral parietal regions^35^, and a disruption in DMN connectivity has been observed in patients following chemotherapy, which was related to cognitive deficits^34^,^36^. Previous neuroimaging studies have demonstrated that breast cancer patients with chemotherapy had disturbed functional and structural networks, which reduced cognitive function. Our findings based on cerebral perfusion changes presented the existence of chemotherapy-induced attention deficits in the current study. As mentioned above, the ANT task is used to examine individual differences in the three attention networks. Thus, we hypothesized that the changes in brain networks for breast cancer patients receiving neoadjuvant chemotherapy could contribute to the attention network deficits and other cognitive impairments. Our data appear to support these hypotheses. The current study shows that after neoadjuvant chemotherapy, patients had significant increases in cerebral perfusion in the left PCC and the superior and inferior frontal, precuneus, and bilateral temporal and parietal regions compared to before chemotherapy, in addition to the right superior frontal regions when compared to healthy controls. Furthermore, the extent of regions with a segmental increase correlated with a decrease in the performance of the attention network. Interestingly, the brain regions that increased consisted of a brain network for attention function that particularly involved the DMN. Our findings suggest that neoadjuvant chemotherapy influences the hemodynamic activity in these brain networks through increasing cerebral perfusion, which reduced performance in attention networks in breast cancer patients.

Several limitations of the present study should be considered. First, the design of this study lacks the matched interval time for healthy controls to eliminate time factors that could affect brain structure and function. Previous studies show that changes in cognitive function, including those related to the ANT task, do not exist in short period intervals for healthy persons^37^,^38^. Furthermore, Nudelman *et al*. showed that the healthy controls showed no change in cerebral perfusion using the ASL when the inter-scan interval was approximately 160 days^15^. Therefore, it was appropriate to use similar controls for comparison to explore the effects of neoadjuvant chemotherapy on cerebral perfusion and attention function. Second, for the current study, we have eliminated the effects of chemotherapy-induced acute symptoms such as fatigue, vomiting, and anemia, which are involved in cognitive functioning. However, it is difficult to eliminate the potential effects of other factors, for example, the use of glucocorticoids and antiemetic drugs^39^,^40^, which could also influence cognitive functioning across the blood brain barrier. Thus, future studies should control for these factors when examining cognitive function to clarify the neural mechanisms of chemotherapy-induced cognitive impairment. Third, there is one methodological limitation that should be considered when interpreting our findings. Our results supported indirectly the existence of chemotherapy-induced attention deficits based on cerebral perfusion changes in this study. Future studies should investigate the underlying neural mechanisms of attention networks deficits more directly by combining ASL functional MRI techniques during attention tasks.

In conclusion, our study represents a significant step toward understanding potential neoadjuvant chemotherapy-induced changes in the attention network and cerebral perfusion. Our findings demonstrated that the neoadjuvant chemotherapy-induced breast cancer patients exhibited impairment of attention networks, which includes the reduced performance in the alerting and executive control attention networks and an increase in the perfusion of specific brain regions. We also reveal the reduced performance in the attention networks, which was correlated with an increase in CBF in related brain regions.

## Materials and Methods

### Participants

The present study was approval from the Research Ethics Committee of the First Affiliated Hospital of Anhui Medical University. We recruited a total of 33 young women with middle-to-late-stage breast cancer who were scheduled to receive neoadjuvant chemotherapy. The patients received preoperative systemic therapy with AC (doxorubicin/cyclophosphamide) followed by preoperative docetaxel for 4 cycles after breast cancer diagnosis. The dosing schedules for AC were followed by docetaxel chemotherapy. Specifically, doxorubicin at 60 mg/m intravenously (IV) was given on day 1, and cyclophosphamide at 600 mg/m IV was given on day 1, which were cycled every 21 days for 4 cycles. Subsequently, docetaxel treatment at 100 mg/m IV was given on day 1 and cycled every 21 days for 4 cycles. Pre-treatment assessment was conducted after diagnosis but before neoadjuvant chemotherapy. Follow-up post-treatment assessment was performed within 1 month after completing eight cycles of neoadjuvant chemotherapy. Thirty-five matched healthy controls participated in the present study. There was only a single assessment for these healthy controls. The detailed information recorded from each participant is described in Table 1. For these participants, The inclusion and exclusion criteria were described in our previous study^6^. Following an explanation of the study objective, informed consent was obtained from all individual participants included in the study. The study was conducted according to the Strengthening the Reporting of Observational Studies in Epidemiology reporting guidelines.

### Neuropsychological background tests and ANT task

As in our previous studies, neuropsychological background tests and the ANT task were assessed for all patients and healthy controls. General cognitive functions were assessed using the Beijing version of the Montreal Cognitive Assessment (MoCA) test. The digit span test was used to measure attention. The Hamilton Depression Rating Scale (HAMD) and HAMA were used to assess participants’ possible depressive and anxiety symptoms. The Stroop test, Trail Making test and Verbal fluency test were used to measure executive function. The Chinese version of the Auditory Verbal Learning Test (AVLT) was used to assess memory. The ANT task^41^, as described in our previous cross-sectional study, is usually used to investigate alerting, orienting, and executive functions in the attention networks. Briefly, lower alerting and orienting network scores indicate reduced performance in the alerting and orienting networks, but higher executive control network scores indicate reduced conflict resolving capabilities. Neuropsychological evaluation and magnetic resonance imaging scans were performed on the same day.

### MRI scan acquisition

All imaging data were acquired on a 3.0-T MR system (Discovery MR750 W, General Electric, Milwaukee, WI, USA) at the First Affiliated Hospital of Anhui Medical University. Multiple modalities included 3D T1-weighted, T2-weighted turbo spin echo (TSE), fluid attenuated inversion recovery (FLAIR) images, and whole brain 3D pseudo-continuous ASL (pcASL) (see details in Supplementary Materials).

### Image analysis

All image data were preprocessed using customized scripts of FSL (FMRIB Software Library, the Analysis Group, FMRIB, Oxford, UK). A voxelwise two-sample t-test was performed using AFNI (Medical College of Wisconsin, Milwaukee, Wisconsin, USA) between every two of the three groups except for the comparison between after treatment and before treatment, where a paired t-test was used instead. The multiple comparison issue was addressed at the cluster level using false discovery rate (FDR) correction, with q < 0.01. A minimum voxel size of 100 was set to exclude small clusters (see details in Supplementary Materials).

### Statistical analysis

SPSS 19.0 (SPSS, Chicago, IL, USA) was used to analyze the clinical and demographic data. Paired *t-*tests were used to assess perfusion change scores and changes in neuropsychological test performance between pre- and post-treatment within the breast cancer patient group. To compare with the healthy controls, independent two-sample t-tests were used to assess the differences in these variables. Because the breast cancer patients in the post-treatment group had lower HAMA scores and higher CRF scores compared with the pre-treatment group, we included the variable as a covariate. We also subtracted pre- from post-treatment scores of the attention networks and CBF values to obtain a change score for each chemotherapy-treated individual. A partial correlation analysis with HAMA and CRF scores as the nuisance covariates was used to assess the relationships between the changes in scores of the attention networks and the changed values of CBF in different regions pre- and post-treatment, with p < 0.05 defined as the significance level.

## Additional Information

**How to cite this article**: Chen, X. *et al*. The attention network changes in breast cancer patients receiving neoadjuvant chemotherapy: Evidence from an arterial spin labeling perfusion study. *Sci. Rep.* **7**, 42684; doi: 10.1038/srep42684 (2017).

**Publisher's note:** Springer Nature remains neutral with regard to jurisdictional claims in published maps and institutional affiliations.

## Supplementary Material

Supplementary Materials

## Figures and Tables

**Table 1:** Demographic characteristics and summary of neuropsychologic test.

	Healthy Controls (n = 34)	Pre-treatment (n = 31)	Post-treatment (n = 31)	P-values	
	Mean(SD)	Mean(SD)	Mean(SD)	Pre vs. Post^a^	HC vs. Post^b^
Age at Baseline (years)	46.29 (4.09)	47.10 (4.53)	NA	0.456	NA
Education (years)	11.21 (2.09)	10.65 (2.36)	NA	0.313	NA
Breast cancer stage					
II	NA	10	NA	NA	NA
III	NA	17	NA	NA	NA
Inter-scan interval (days)	NA	NA	166.61 (4.46)	NA	NA
Fatigue	19.82 (3.87)	21.19 (3.02)	24.71 (3.12)	<0.001*	<0.001*
HAMA	3.97 (1.22)	5.19 (1.05)	4.23 (0.96)	0.003	0.354
HAMD	4.38 (1.28)	4.94 (0.93)	4.52 (1.24)	0.114	0.670
MoCA	25.50 (1.93)	24.74 (2.22)	25.39 (2.33)	0.299	0.832
Attention/concentration					
Digit Span (forward)	6.12 (1.27)	5.74 (1.24)	5.61 (1.02)	0.670	0.085
Digit Span (backward)	5.24 (1.10)	4.87 (1.09)	4.16 (0.86)	0.010	<0.001*
Stroop Color test (sec)	14.43 (4.17)	15.16 (3.25)	15.11 (3.45)	0.955	0.483
Trailmaking A (sec)	52.88 (9.22)	53.63 (9.51)	56.64 (8.14)	0.175	0.087
Memory (AVLT)					
Immediate Recall	11.44 (1.76)	11.13 (1.73)	10.90 (1.51)	0.607	0.193
Delayed Recall	10.56 (1.93)	10.23 (1.93)	8.84 (1.61)	0.013	<0.001*
Recognition	9.62 (2.16)	9.58 (1.84)	7.87 (1.91)	0.005	0.001*
Executive function					
Trailmaking B (sec)	97.02 (9.85)	96.68 (9.15)	102.38 (10.36)	0.016	0.036
Stroop Word test (sec)	17.43 (2.47)	18.38 (3.78)	22.20 (6.19)	0.010	<0.001*
Stroop Interference test (sec)	30.98 (8.38)	31.98 (7.96)	36.82 (9.44)	0.022	0.010
Verbal fluency	13.15 (2.12)	12.87 (1.46)	12.26 (1.88)	0.178	0.080

Abbreviations: SD, standard deviation; HC, Healthy Controls; HAMA, Hamilton Anxiety Rating Scale; HAMD, Hamilton Depression Rating Scale; MoCA, Montreal Cognitive Assessment Test; AVLT: Auditory Verbal Learning Test.

^a^Paired t-tests.

^b^Independent two-sample t-tests.

^*^Indicates significance at FDR corrected for multiple comparisons.

**Table 2:** Attention network scores of breast cancer patients and healthy controls.

	Healthy Controls (n = 34)	Pre-treatment (n = 31)	Post-treatment (n = 31)	P-values	
	Mean RT(ms) (SD)	Mean RT(ms) (SD)	Mean RT(ms) (SD)	Pre vs. Post^a^	HC vs. Post^b^
Alerting	32.21 ± 16.97	31.19 ± 18.81	19.94 ± 18.83	0.038	0.007*
Ratio	0.051 ± 0.028	0.049 ± 0.031	0.030 ± 0.029	0.025	0.005*
Orienting	55.79 ± 22.30	62.68 ± 31.25	64.58 ± 27.64	0.701	0.162
Ratio	0.090 ± 0.040	0.097 ± 0.046	0.098 ± 0.041	0.981	0.441
Executive	94.18 ± 29.01	97.65 ± 30.08	110.29 ± 34.21	0.006	0.044
Ratio	0.150 ± 0.048	0.154 ± 0.051	0.168 ± 0.054	0.044	0.169
Mean RT	630.97 ± 60.31	643.81 ± 72.39	666.16 ± 72.81	0.002*	0.037
Accuracy (%)	98.44 ± 1.26	98.58 ± 1.03	98.23 ± 1.23	0.062	0.489

Abbreviations: SD, standard deviation; HC, Healthy Controls; RT, reaction time.

^a^Paired t-tests.

^b^Independent two-sample t-tests.

^*^Indicates significance at FDR corrected for multiple comparisons.

**Table 3:** Comparison of CBF values (ml/100 g / min) of corresponding parts for all participants.

Region (CBF values)	Healthy Controls (n = 34)	Pre-treatment (n = 31)	Post-treatment (n = 31)	*P*-values	
	Mean(SD)	Mean(SD)	Mean(SD)	Pre vs. Post^a^	HC vs. Post^b^
Left middle cingulate gyrus	61.90 (7.63)	NA	54.35 (8.64)	NA	0.015
Left posterior cingulate gyrus	65.39 (9.83)	67.19 (9.40)	74.79 (14.55)	0.003	0.004
Left superior frontal gyrus	NA	47.70 (6.27)	51.63 (7.73)	0.006	NA
Right superior frontal gyrus	49.55 (6.43)	NA	54.35 (8.64)	NA	0.013
Left inferior frontal gyrus	NA	54.82 (7.11)	59.47 (8.93)	0.002*	NA
Left superior parietal gyrus	45.60 (6.22)	NA	50.25 (8.45)	NA	0.013
Right superior parietal gyrus	NA	50.30 (7.68)	54.20 (9.31)	0.020	NA
Inferior parietal gyrus	51.26 (5.83)	51.44 (7.46)	57.33 (9.55)	0.001*	0.004
Superior occipital gyrus	45.05 (6.07)	46.52 (7.71)	51.35 (8.82)	0.002*	0.001*
Left middle occipital gyrus	45.97 (4.73)	47.53 (7.65)	53.15 (9.32)	0.003	<0.001*
Inferior occipital gyrus	49.18 (5.09)	NA	56.02 (9.46)	NA	0.001*
Superior temporal gyrus	53.18 (5.23)	52.76 (7.24)	59.23 (8.08)	<0.001*	0.001*
Middle temporal gyrus	52.55 (5.47)	52.28 (7.23)	58.09 (8.77)	<0.001*	0.004
Inferior temporal gyrus	50.47 (4.77)	51.01 (7.28)	56.57 (8.51)	0.001*	0.001*
Precentral gyrus	50.71 (6.04)	51.61 (6.20)	55.93 (8.64)	0.004	0.006
Supramarginal gyrus	52.45 (5.32)	52.99 (7.67)	58.62 (9.34)	<0.001*	0.002*
Angular gyrus	50.36 (5.36)	50.51 (7.85)	56.82 (9.93)	0.002*	0.002*
Precuneus	57.24 (8.70)	60.20 (8.88)	64.75 (11.68)	0.014	0.004
Cuneus	50.77 (7.96)	53.49 (8.08)	59.17 (10.25)	0.003	<0.001*
Calcarine cortex	52.25 (7.16)	54.37 (7.14)	60.32 (9.91)	0.002*	<0.001*
Lingual gyrus	50.50 (6.54)	NA	58.09 (8.38)	NA	<0.001*
Fusiform gyrus	45.41 (4.66)	NA	51.03 (7.24)	NA	0.001*

Abbreviations: CBF, cerebral blood flow; SD, standard deviation; HC, Healthy Controls; NA, not applicable.

^a^Paired t-tests.

^b^Independent two-sample t-tests.

^*^Indicates significance at FDR corrected for multiple comparisons.
