# Supplementary material for: The attention network changes in breast cancer patients receiving neoadjuvant chemotherapy: Evidence from an arterial spin labeling perfusion study
Source: Sci Rep. 2017 Feb 17;7:42684. doi: 10.1038/srep42684 (PMC5314322; doi:10.1038/srep42684)
Supplement: Supplementary Materials [file srep42684-s1.pdf]

The attention network changes in breast cancer patients receiving neoadjuvant chemotherapy: Evidence from an arterial spin labeling perfusion study

Xingui Chen [1,2,3,*], Xiaoxuan He[4,*], Longxiang Tao[5], Huaidong Cheng[6], Jingjing Li[6], Jingjie Zhang [7], Bensheng Qiu[4], Yongqiang Yu[5] & Kai Wang[1,2,3]

[1]Department of Neurology, The First Affiliated Hospital of Anhui Medical University, Hefei, China;

[2]Department of Medical Psychology, Anhui Medical University, Hefei, China;

[3]Collaborative Innovation Centre of Neuropsychiatric Disorders and Mental Health, Anhui Province, China;

[4]Center for Biomedical Engineering, University of Science and Technology of China, Hefei, China;

[5]Department of Radiology, The First Affiliated Hospital of Anhui Medical University, Hefei, China;

[6]Department of Oncology, The Second Affiliated Hospital of Anhui Medical University, Hefei, China;

[7]Department of Breast Surgery, The First Affiliated Hospital of Anhui Medical University, Hefei, China;

Supplementary Methods

MRI scan Acquisition

All participants underwent fMRI scans at the First Affiliated Hospital of Anhui Medical University. Two scans were performed before neoadjuvant chemotherapy and within 1 month after completing chemotherapy for the breast cancer patients. Healthy controls underwent only a single fMRI scan. We instructed all participants during the scan to keep their eyes closed, think of nothing in particular, relax and move as little as possible, and to not fall asleep.

The MRI was performed using a 3.0-T MR system (Discovery MR750 W, General Electric, Milwaukee, WI, USA). Tight but comfortable foam padding was used to minimize head motion, and earplugs were used to reduce scanner noise. The T1-weighted 3D-SPGR images were obtained with the following parameter settings: repetition time (TR) = 7.2 ms; echo time (TE) = 3.1 ms; flip angle = 12; slice thickness = 1 mm; image resolution = 1 mm × 1 mm × 1 mm; field of view (FOV) = 24 cm × 24 cm; matrix size = 256 × 256; acquisition time approximately 9 min; and 172 sagittal slices prescribed to cover the entire brain. Subsequently, resting-state perfusion imaging was performed using whole brain three-dimensional (3D) pseudo-continuous ASL (pCASL) that focused on quantitative signal targeting using alternating radio frequency pulse labeling of arterial regions (QUASAR). The imaging parameters were as follows: TR = 5,633 ms; TE = 13 ms; post-label delay = 2,025 ms; spiral in readout of 8 arms with 512 sample points; flip angle = 111; FOV = 24 cm × 24 cm; slice thickness = 2 mm; matrix size = 128 × 128; image resolution = 2 mm × 2 mm × 2 mm; and 80 axial slices prescribed to cover the entire brain. For the resting state ASL data, the acquisition time was 5 min and 27 s. The original images of 3D ASL were retrieved from a commercially available system (GE Advantage Windows [AW] Workstation, version 4.2 using Functool software, Milwaukee, WI), and CBF maps

were also automatically generated. During the ASL scans, all subjects were instructed to keep their eyes closed, relax and move as little as possible, think of nothing in particular, and to not fall asleep. The T2-weighted TSE (19 transversal slices, 240*240-mm^2 FOV, 5-mm slice thickness, 5,290-ms TR, and 120-ms TE), and a FLAIR (19 transversal slices; 240*240-mm^2 FOV, 136.6-ms TE, 9,000-ms TR, 5-mm slice thickness, and 256*256 matrix) were also acquired to search for a primary brain pathology as an exclusion criterion.

Image Analysis

All image data were preprocessed using customized scripts of FSL (FMRIB Software Library, the Analysis Group, FMRIB, Oxford, UK). Initially, an intensity bias field correction was applied to the FSPGR T1W images, followed by brain extraction and normalization into the Montreal Neurological Institute standard space. To achieve the best registration results, each subject's T1W volume was co-registered to ASL volumes. Visual inspection was performed for each subject to identify the best registered outcome, using the transformation matrix to further register the CBF volume to the T1W volume. Thereafter, the registered CBF volume was normalized into 2 x 2 x 2 mm^3 and smoothed with a Gaussian FWHM kernel of 6 x 6 x 6 mm^3. After this preprocessing, all CBF volumes of all subjects were ready for statistical analysis.

A voxelwise two-sample t-test was performed using AFNI (Medical College of Wisconsin, Milwaukee, Wisconsin, USA) between every two of the three groups except for the comparison between after treatment and before treatment, where a paired t-test was used instead. A Gray Matter mask was applied to remove regions including white matter and ventricles. The multiple comparison issue was addressed at the cluster level using false discovery rate (FDR) correction, with $q < 0.01$. A

minimum voxel size of 100 was set to exclude small clusters. To identify the atlases of the clusters, a customized MATLAB script was written to locate each cluster in the Anatomical Automatic Labeling (AAL) template and the coordinates of its peak. These atlases were then selected as regions of interest, and the mean CBF values were extracted using WFU PickAtlas (Wake Forest University, Winston-Salem, NC) for each subject. The individual CBF value in these atlases was then used for intergroup statistical analysis.

Supplementary Results

Table S1. Patients with post-treatment have greater perfusion than patients with pre-treatment

Regions (hemisphere)		Atlas location of peak t value			Cluster Size	Peak t values[b]
		x[a]	y[a]	z[a]		
Posterior cingulate gyrus	L	-2	34	28	152	4.12
Superior frontal gyrus	L	-22	-16	48	112	4.30
Inferior frontal gyrus	L	-36	-32	2	155	4.61
Superior parietal gyrus	R	36	56	58	208	4.59
Inferior parietal gyrus	L	-48	50	36	642	5.16
	R	32	52	48	102	4.59
Superior occipital gyrus	L	-22	80	22	212	6.41
	R	20	80	30	115	4.35
Middle occipital gyrus	L	-24	82	18	315	7.14
Superior temporal gyrus	L	-52	20	2	820	5.63
	R	64	0	-6	874	6.56
Middle temporal gyrus	L	-64	8	-22	909	6.47
	R	52	56	16	791	5.15
Inferior temporal gyrus	L	-64	10	-26	217	5.47
	R	58	44	-24	328	4.92
Precentral gyrus	L	-54	2	22	470	5.85
	R	64	-6	4	172	5.28
Supramarginal gyrus	L	-48	48	34	244	5.27
	R	58	32	28	258	4.99
Angular gyrus	L	-40	66	50	212	5.33
	R	44	66	30	426	4.95
Precuneus	L	-10	56	32	145	4.36
	R	14	50	14	459	4.73
Cuneus	L	-10	88	22	114	4.92
	R	14	78	32	226	4.81
Calcarine cortex	L	-10	82	8	238	4.67
	R	16	54	12	104	4.28

Abbreviations: R, Right; L, Left.

[a] MNI coordinates.

[b] All $P_{FDR\text{-}corr} < 0.01$, $p < 0.0027$.

Table S2. Healthy controls have lesser perfusion than patients with post-treatment

Regions (hemisphere)		Atlas location of peak t value			Cluster Size	Peak t values[b]
		x[a]	y[a]	z[a]		
Middle cingulate gyrus	L	-8	-12	34	175	-4.43
Posterior cingulate gyrus	L	-10	44	30	121	-3.83
Superior frontal gyrus	R	16	-28	48	153	-4.28
Superior parietal gyrus	L	-24	60	44	237	-4.15
Inferior parietal gyrus	L	-34	62	44	480	-4.12
	R	42	42	44	596	-4.19
Superior occipital gyrus	L	-14	94	2	335	-4.60
	R	20	72	28	118	-3.67
Middle occipital gyrus	L	-14	94	0	1061	-4.82
Inferior occipital gyrus	L	-28	80	-12	599	-4.61
	R	42	64	-12	120	-4.25
Superior temporal gyrus	L	-44	44	18	429	-4.29
	R	50	42	12	924	-4.71
Middle temporal gyrus	L	-46	52	20	471	-4.76
	R	48	54	-2	808	-5.27
Inferior temporal gyrus	L	-42	48	-14	817	-4.43
	R	42	60	-6	709	-4.93
Precentral gyrus	L	-52	2	26	103	-3.58
	R	40	20	40	239	-3.92
Supramarginal gyrus	L	-50	50	30	225	-4.60
	R	64	34	24	522	-4.49
Angular gyrus	L	-44	50	22	293	-4.38
	R	52	50	30	177	-3.73
Precuneus	L	-2	62	24	857	-4.03
	R	6	72	32	532	-3.98
Cuneus	L	-8	76	28	745	-4.50
	R	4	76	28	595	-4.42
Calcarine cortex	L	-14	94	-2	1010	-4.67
	R	16	74	18	305	-4.59
Lingual gyrus	L	-18	88	-18	939	-5.48
	R	2	74	-4	506	-4.97
Fusiform gyrus	L	-20	86	-18	638	-5.35
	R	32	50	-20	661	-5.30

Abbreviations: R, Right; L, Left.

[a] MNI coordinates.

[b] All $P_{FDR\text{-}corr} < 0.01$, $p < 0.0046$.
